# Identification and Validation of MTFP1 as a Mitochondrial Target Restoring Dynamics and ECM Remodeling in Acute Myocardial Infarction

**DOI:** 10.3390/cimb48030293

**Published:** 2026-03-09

**Authors:** Xi Hu, Hailong Bao, Yue Huang, Zhaoxing Cao, Wei Yang, Cheng Huang, Xin Chen, Yanbing Chen, Bingxiu Chen, Guiling Xia, Xiao Yang, Runze Huang, Zhangrong Chen

**Affiliations:** 1 Department of Cardiovascular Medicine, The Affiliated Hospital of Guizhou Medical University, Guiyang 550004, China; 17685300286@163.com (X.H.); baohailong@stu.gmc.edu.cn (H.B.); huang_yue@gmc.edu.cn (Y.H.); 17885718526@163.com (C.H.); cbx1905@outlook.com (B.C.); 2 The Key Laboratory of Myocardial Remodeling Research, The Affiliated Hospital of Guizhou Medical University, No. 28 Guiyi Road, Guiyang 550004, China; 15011763972@163.com (Z.C.); 18786089650@163.com (W.Y.); m15730437027@163.com (X.C.); 15985162243@163.com (Y.C.); guiling13984860037@163.com (G.X.); 15004112620@163.com (X.Y.)

**Keywords:** acute myocardial infarction, mitochondria, MTFP1, ECM remodeling, biomarkers, DRP1/MMP9/TIMP1 axis

## Abstract

**Background**: Mitochondrial dysfunction is central to the pathogenesis of acute myocardial infarction (AMI), but mitochondria-related molecular biomarkers and mechanisms remain incompletely defined. This study aimed to identify mitochondria-associated biomarkers in AMI and elucidate their functional roles in mitochondrial dynamics, extracellular matrix (ECM) remodeling, and cardiac protection. **Methods**: Two GEO datasets (GSE19322, GSE71906) were analyzed to identify mitochondria-related differentially expressed genes (DE-MRGs) by intersecting DEGs with MitoCarta3.0 genes. Functional enrichment (GO/KEGG), LASSO regression, ROC curves, and nomogram modeling were employed to screen biomarkers. Immune infiltration profiling, GeneMANIA, GSEA, TF-mRNA and ceRNA network construction, and drug prediction analyses were performed. Expression validation was conducted via RT-qPCR, Western blot (WB), and immunohistochemistry (IHC) in murine AMI models and hypoxia-induced cardiomyocytes. Functional assays assessed cardiac performance (echocardiography), infarct size (TTC staining), fibrosis (Masson/Sirius red), oxidative stress (ROS), and ECM remodeling (MMP9/TIMP1 axis). **Results**: We identified 295 DE-MRGs, enriched in oxidative phosphorylation and mitochondrial metabolic pathways. Machine learning and validation analyses pinpointed MTFP1 and DNAJC28 as AMI biomarkers with strong diagnostic accuracy. In vivo and in vitro studies confirmed marked downregulation of MTFP1 post-AMI and under hypoxia. AAV9-mediated MTFP1 overexpression improved cardiac function, reduced infarct size, attenuated fibrosis, and decreased ROS levels. Mechanistically, MTFP1 upregulated phosphorylated DRP1 (Ser616) without altering total DRP1, balanced MMP9/TIMP1 activity, and suppressed fibrosis markers (COL1A1, α-SMA). Gelatin zymography indicated that MMP9 activation remained restrained despite elevated pro-MMP9, consistent with TIMP1-mediated regulation. Hypoxia-induced cardiomyocytes showed similar antifibrotic and antioxidative responses following MTFP1 overexpression. **Conclusions**: Our study identified MTFP1 as a novel mitochondria-related biomarker and therapeutic modulator in AMI. MTFP1 exerts cardioprotective effects by restoring mitochondrial fission balance and ECM remodeling through the p-DRP1/MMP9/TIMP1 signaling axis, attenuating fibrosis and oxidative stress. These findings provide mechanistic insight into mitochondria-targeted cardioprotection and highlight MTFP1 as a promising diagnostic and therapeutic target for AMI.

## 1. Introduction

AMI is defined as myocardial cell necrosis due to myocardial ischemia, and is often clinically divided into ST-segment elevation myocardial infarction and non-ST-segment elevation myocardial infarction. AMI has become a leading global cause of hospitalization and mortality, posing a severe threat to human health. Its pathogenesis involves rupture of atherosclerotic plaques in coronary arteries, resulting in acute thrombotic occlusion that critically restricts or completely obstructs myocardial blood flow. This leads to myocardial hypoxia and nutrient deprivation, ultimately causing myocardial cell death. Key risk factors include positive early family history, smoking, hypertension, dyslipidemia, and diabetes mellitus. Clinical manifestations predominantly feature severe precordial pain, accompanied by complications such as arrhythmias, fever, tachycardia, gastrointestinal symptoms, and potentially hypotension, shock, or progression to heart failure in advanced stages. The rate of asymptomatic left ventricular systolic dysfunction in patients after acute myocardial infarction is as high as 30–60% [1]. Heart failure (HF) remains a major contributor to disability, symptom burden, and hospital readmissions. Primary treatment for AMI focuses on rapid restoration of coronary blood flow (termed acute myocardial reperfusion) via coronary angioplasty and stent implantation (percutaneous coronary intervention, PCI) to remove thrombotic occlusion and minimize acute ischemic injury to the myocardium. Despite timely PCI, AMI patients continue to face substantial mortality and morbidity, underscoring the need for novel therapeutic targets to protect the myocardium from acute ischemic insults and prevent the onset of HF.

The heart is the organ with the highest energy demand in the entire organism. Mitochondria serve as the primary sites for intracellular energy production, with cardiac mitochondria occupying 40% of myocardial cell volume [2]. They govern cardiomyocyte metabolism and survival [3]. Mitochondria are double-membrane-bound organelles composed of an inner mitochondrial membrane (IMM) and outer mitochondrial membrane (OMM), which separate the intermembrane space (IMS) from the matrix. Functioning as metabolic hubs and signaling platforms, mitochondria participate in critical cellular processes including: ATP generation via oxidative phosphorylation (OXPHOS), fatty acid oxidation, calcium buffering, phospholipid synthesis, ROS production and homeostasis, iron–sulfur cluster biogenesis, and innate immune signaling [4]. Substantial evidence shows that mitochondrial dysfunction plays an important role in the pathogenesis of multiple cardiovascular diseases. Firstly, cardiomyocytes primarily rely on fatty acid-driven OXPHOS for ATP production under physiological conditions [5]. Reduced mitochondrial bioenergetic efficiency directly compromises myocardial contractility. Secondly, as Ca^2+^ flux is central to cardiac activity [6], defective mitochondrial regulation of Ca^2+^ homeostasis (in coordination with the endoplasmic reticulum) may disrupt electrical conduction and cardiac function. Finally, physiological inflammatory homeostasis is particularly important for cardiac function [7], and the accumulation of damaged mitochondria in the cytoplasm of cardiomyocytes or endothelial cells can drive pathogenic inflammatory responses and thus affect cardiac function. During acute myocardial ischemia in AMI patients, oxygen/nutrient deprivation initiates severe biochemical and metabolic disturbances in cardiomyocytes, many of which adversely affect mitochondrial function and ATP production [8]. In addition, these changes in mitochondrial Ca^2+^ overload, oxidative stress, rapid PH correction, and mitochondrial permeability conversion pores (MPTP) opening exacerbate the harmful effects caused by acute myocardial ischemia. Therefore, exploring mitochondria-related molecular targets in acute myocardial infarction can provide a new therapeutic strategy for the clinical treatment of acute myocardial infarction.

Microarray analysis utilizing differentially expressed genes (DEGs) may exhibit limitations in reproducibility and sensitivity [9,10]. Machine learning can improve the prediction and accuracy of these key genes identified using conventional microarrays or next-generation sequencing data [11]. The most widely employed machine learning techniques include Least Absolute Shrinkage and Selection Operator (LASSO) regression and Support Vector Machine-Recursive Feature Elimination (SVM-RFE) algorithms [12]. This study aims to screen mitochondria-related biomarkers in AMI using bioinformatics approaches and construct a nomogram model for predicting individual AMI probability. Comprehensive analyses of these biomarkers provide theoretical insights into mitochondrial roles in AMI pathogenesis. Furthermore, pharmacological prediction of candidate drugs targeting mitochondria-related AMI offers a foundation for clinical therapeutics. Our analytical framework encompasses four dimensions: (1) Identification of mitochondria-related DEGs in AMI using the “limma” R package, supplemented by GO and KEGG pathway enrichment analyses. (2) Large-scale screening and diagnostic validation of AMI-associated molecular biomarkers through machine learning methodologies. (3) Experimental validation of gene expression levels in a murine myocardial infarction model. (4) The biological function and possible mechanism of action of MTFP1 were identified through genetic manipulation tools. This integrated approach systematically explores mitochondrial contributions to AMI progression while identifying potential therapeutic targets.

In this study, we describe the pathway regulated by MTFP1, which improves mitochondrial fission balance and ECM remodeling, helps to alleviate myocardial fibrosis and oxidative stress, thus protecting cardiac function and improving prognosis. Overexpression of MTFP1 significantly increased the level of p-DRP, which regulated the balance between mitochondrial fusion and division. Mitochondrial dynamics is a steady-state balance between mitochondrion fission and fusion, which is controlled by two different kinds of protein [13]. Mitochondrial fusion involves mitochondrial fusion proteins 1 and 2 (MFN1/2) in the outer membrane of mitochondria and optic atrophy 1 (OPA1) in the inner membrane of mitochondria, while dynamic related protein 1 (DRP1) is considered as the key protein of mitochondrial fission, and additionally, MTFP1 is also involved in mitochondrial fission [14]. MTFP1 activity in mitochondrial fission is mediated by essential fission GTP enzyme DRP1. Mitochondrial debris activates extracellular signal transduction, so we observed a significant upregulation of MMP9 and TIMP1. Matrix metalloproteinases (MMP) are proteases involved in the degradation of ECM, and the activity of MMP is accurately regulated at the transcription level and the activation level of precursor Zymogens [15]. Tissue inhibitors of metalloproteinases (TIMP) are specific inhibitors of MMP involved in local activities in tissues [16]. The balance of extracellular matrix depends largely on the close interaction between MMP and TIMP. Our results show that although the synthesis of MMP9 is enhanced, its activation is effectively limited, which may be due to the simultaneous induction of TIMP1, but it can still maintain a balanced ECM proteolysis. Therefore, MTFP1 can maintain ECM steady-state through the regulation of MMP9 activity mediated by TIMP1.

## 2. Materials and Methods

**Online data acquisition and processing**

The AMI datasets (GSE19322 and GSE71906) were obtained from GEO database (https://www.ncbi.nlm.nih.gov/, accessed on 8 August 2023). Infarct tissue samples from 14 healthy control mice and 16 AMI mice were screened from the GSE19322 dataset and subjected to gene microarray assay using the GPL339 platform, which served as a training set for subsequent analyses. Testing set of GSE71906 contains cardiac samples from six healthy control mice and six AMI mice that were subjected to gene microarray assays using the GPL8321 platform. The 1140 MRGs were taken from the MitoCarta3.0 database (http://www.broadinstitute.org/mitocarta, accessed on 8 August 2023).

**DEGs and DE-MRGs identification and functional enrichment**

In the GSE19322 dataset, DEGs was performed on the 16 disease samples and 14 control samples using the “limma” R package (version 3.42.2) [17]. The thresholds were set at *p* value < 0.05 and |log2fold change (FC)| > 1. A volcano map and a heatmap were plotted using the “ggplot2” R package (version 3.3.2) [18] and the “complexheatmap” R package (version 2.14.0) [19], respectively, taking the intersection of the above DEGs as well as the 1140 MRGs to get the DE-MRGs.

The “clusterProfiler” R package (version 3.14.3) [20] was used to analyze the GO system and KEGG pathway enrichment analysis of DE-MRGs, with the significance threshold set at *p* value < 0.05 and the results were visualized by “ggplot2” R package (version 3.3.2) [18].

**Machine learning for screening biomarkers**

Firstly, signature genes were screened by LASSO regression analysis used “glmnet” R package (family = “binomial”, type. Measure = “class”, nfold = 10) (version 4.0-2) [21]. In addition, the expression amounts of the signature genes were extracted from the GSE19322 and GSE71906 respectively and the expression of signature genes was plotted using the “ggplot2” (version 3.3.2) [18] combination with the sample grouping information from the respective datasets. Finally, the signature genes with consistent and significant expression in the two datasets were identified as biomarkers.

To demonstrate that biomarkers could distinguish between diseased and normal samples, we validated the biomarkers using an artificial neural network (ANN) by “neuralnet” R package (version 3.4.2) [22]. In addition, ROC curve was performed to evaluate the predictive performance of AMI classification models in the GSE19322 and GSE71906 datasets.

**Nomogram construction**

In the GSE19322 dataset, based on the biomarkers obtained from the above analysis, information of the sample grouping and biomarkers expression was combined, and we used the “RMS” R package (version 6.2-0) [23] to construct a nomogram model. Each biomarker in the model corresponds to a score, which was summed to get the total score and determined the diagnostic effectiveness of AMI. In addition, the calibration curve and ROC curve were plotted to verify the validity of the nomogram.

**Functional analysis of biomarkers**

To understand the functions and related proteins of these biomarkers, we utilized the GeneMANIA database (http://genemania.org/, accessed on 20 August 2023) to construct the GeneMANIA Network. Furthermore, in order to investigate the potential functions of the biomarkers, in the GSE19322 dataset, we calculated the Spearman correlation between the biomarkers and all other genes, and then sorted the results. Next, the “mh.all.v2023.1.Mm.symbols.gmt” gene set from the MSigDB database was selected as the background set for GSEA of the biomarkers.

**Immune infiltration analysis and drug prediction of biomarkers**

To explore immune infiltration in AMI, we firstly performed an immune cell analysis on samples from the AMI and control samples of the GSE19322 dataset using the CIBERSORT algorithm to assess the proportion of immune cells in each sample (*p* < 0.05). Second, we used Wilcoxon to test whether immune cells were significantly different in the AMI patient and control sample groups and obtained differential immune cells. Finally, the correlation between biomarkers and differential immune cells was calculated by Spearman and presented in a heatmap. In addition, the drug signatures database (DSigDB) from Enrichr platform had been used to predict the drugs that interact with biomarkers.

**TF-mRNA and ceRNA network construction**

Transcription factors (TFs) of biomarkers were predicted by using the miRNet platform (an miRNA-centric network visual analytics platform) based on the ENCODE database (https://www.encodeproject.org/, accessed on 28 August 2023), the JASPAR database (http://jaspar.genereg.net/, accessed on 30 August 2023) and the RegNetwork database (https://www.regnetworkweb.org/, accessed on 30 August 2023).

The miRNA prediction analysis of biomarkers was performed using the NetworkAnalyst database (https://www.networkanalyst.ca, accessed on 31 August 2023) with organism was mouse and Gene-miRNA interaction TarBase v8.0 database (https://www.tarbase.com/, accessed on 31 August 2023), the miRNA-mRNA were obtained. In addition, miRNA nodes that interact with both biomarkers was screened for. Next, the LncBase database (https://diana.e-ce.uth.gr/lncbasev3/interactions, accessed on 31 August 2023) was utilized to predict the target lncRNAs of the resulting miRNAs and lncRNAs were screened with support from more than two publications. Finally, the mRNA-miRNA-lncRNA regulatory network was mapped by Cytoscape software (v3.10.0).

**Animal information**

Animal experiments were conducted according to the management and use standards of experimental animals in Guizhou Medical University. Male C57BL/6 mice (6–8 weeks old) were purchased from Beijing Weitong Lihua Company (Beijing, China). All animals were kept under the specific pathogen-free (SPF) environment and maintained in a temperature-controlled room (22 °C ± 1 °C) with a 12 h light/dark cycle and 40–60% humidity with ad libitum access to food and water.

**Animal model establishment**

According to the method described before [24], in order to establish an acute myocardial infarction model in mice, mice aged 6–8 weeks were randomly divided into the following groups: Sham-operated group (*n* = 3): Mice were anesthetized with pentobarbital sodium (30 mg/kg), fixed in a supine position, and ECG was monitored after tracheal intubation. A thoracotomy was performed without ligation of the left anterior descending coronary artery (LAD). MI group (*n* = 3): After thoracotomy, the LAD was ligated with a 5-0 suture, and successful occlusion was confirmed by ST-segment elevation on ECG. Heart tissues were harvested for subsequent experiments on the 7th day post-surgery. All animal experimental protocols are approved by the Animal Care and Use Committee of Guizhou Medical University. The study strictly adhered to the recommendations of the Guide for the Care and Use of Laboratory Animals by the National Institutes of Health. The research protocol was approved by the Animal Experimental Ethics Committee of Guizhou Medical University (date of approval: 15 December 2025; NO: 2503640).

**Cell culture**

The H9c2 cell line was utilized as an experimental model. Cells were cultured in DMEM medium (Gibco, ThermoFisher, Waltham, MA, USA). containing 10% fetal bovine serum (vivacell, Shanghai, China), streptomycin (100 μg/mL) and penicillin (100 U/mL) and cultured in a humidified incubator (37 °C) containing 5% CO_2_. All experiments utilized cells in the logarithmic growth phase to ensure consistency and reliability for subsequent cellular assays.

**Cell hypoxia model**

To simulate the myocardial hypoxia model in mouse, H9c2 cells were randomly inoculated into wells (1 × 10^5^ cells per well) after 80–90% fusion. Then, the cells were cultured in a three gas incubator (37 °C, 5% CO_2_, 3% O_2_) for 12 h, 24 h or 36 h. H9C2 cells in control group were cultured in normoxic incubator (37 °C, 5% CO_2_, 21% O_2_).

**Engineering and Transfection of AAV9-MTFP1 Viral Vector**

Recombinant AAV9 vectors carrying the mouse *MTFP1* gene (AAV9-*MTFP1*) were produced in HEK293T cells using a triple-plasmid system with an MTFP1 shuttle vector, ΔF6 helper plasmid, and RC2/9 capsid plasmid. Cells at ~80% confluence in ten 15 cm dishes were transfected with plasmids (70 μg shuttle, 200 μg ΔF6, 70 μg RC2/9) using PEI, followed by medium replacement after 16 h and harvest at 60 h. Cells were lysed by freeze–thaw cycles, and viral particles were purified via iodixanol gradient ultracentrifugation and concentrated using Amicon filters (Millipore, Sigma, Burlington, MA, USA). Viral titers (3–5 × 10^13^ vg/mL) were quantified by qPCR, aliquoted, and stored at −80 °C for intramyocardial injection, with AAV9-GFP prepared as control. For in vivo viral administration, mice were randomly allocated to receive AAV9-*MTFP1* or AAV9-GFP using a computer-generated randomization schedule. Viral aliquots were prepared and labeled with anonymized codes by an independent investigator, such that personnel performing injections, tissue collection, and downstream outcome assessments were blinded to treatment assignment. Group codes were disclosed only after all analyses were finalized.

**Real-time quantitative polymerase chain reaction (RT-qPCR)**

Total RNA was extracted from mouse heart tissue using the Total RNA Kit I (Omega, Norcross, GA, USA). The integrity and purity of total RNA was detected using an ultramicro spectrophotometer (Shenhua Technology, Hangzhou, Zhejiang Province, China). Total RNA was reverse transcribed into cDNA using PrimeScript RT reagent Kit with gDNA Eraser (Takara Bio, Shiga, Japan). Real-time quantitative PCR (RT-qPCR) was performed on a real-time PCR system (Tianlong, Xi’an, Shanxi Province, China) with TB Green Premix EX Taq™ II (TaKaRa Bio, Shiga, Japan). Relative fold changes in mRNA expression levels of target genes across different samples were determined using the 2−ΔΔCt method, with actin serving as the internal reference.

The *MTFP1* primers used in this study were as follows:

Forward(F): 5′-AGGCTTCACCATCAACCGTC-3′,

Reverse(R): 5′-GTCCAAGTGTGGTGGTGGTC-3′.

**Protein extraction and Western blot**

Myocardial tissue was lysed with NP-40 lysate (Solaibo, N8032, Beijing, China) on ice for 30 min, and centrifuged at 10,000× *g* at 4 °C for 10 min. The protein concentration in the supernatant was quantified using a BCA protein assay kit (YaMei, Shanghai, China) and protein in supernatant was heated at 95 °C for 10 min. A total of 35 ug of protein samples were separated by SDS-polyacrylamide gel electrophoresis (SDS-PAGE), and transferred onto a nitrocellulose (NC) membrane (millpore, Billerica, Germany, 0.22 μm). The membrane was blocked with 5% skim milk at room temperature for 1 h, washed three times with TBST, and incubated overnight at 4 °C with primary antibodies. MTFP1 (Proteintech, Wuhan, Hubei Province, China; Cat# 14257-1-AP; dilution 1:1000), β-actin (Proteintech; Cat# 66009-1-Ig; dilution 1:5000). DRP (CST, Danvers, MA, USA; Cat# 8750; dilution 1:1000). P-DRP (CST; Cat# 3455; dilution 1:1000). MMP9 (Proteintech; Cat# 10375-2-AP; dilution 1:1000). TIMP1 (Proteintech; Cat#16644-1-AP; dilution 1:1000). α-SMA (Proteintech; Cat# 14395-1-AP; dilution 1:10,000). α-SMA (Proteintech; Cat# 14695-1-AP; dilution 1:2000). The membrane was incubated with corresponding HRP-conjugated secondary antibodies at room temperature for 1 h. Goat anti-mouse IgG (Proteintech; Cat# SA00001-1; dilution 1:10,000–15,000), goat anti-rabbit IgG (Proteintech; Cat# SA00001-2; dilution 1:10,000–15,000), followed by three washes with TBST. Protein bands were visualized using an ECL kit (Meilun, Dalian, Liaoning Province, China) and imaged.

**Echocardiography measurement**

After 7 days post-MI surgery, a two-dimensional transthoracic echocardiographic examination was performed. The ultrasound assessment was conducted using an ultrasound imaging system (Model: SiliconWave 60; Cona Medical Technology Co., Ltd., Suzhou, Jiangsu Province, China) and key cardiac parameters, including ejection fraction (EF), left ventricular shortening fraction (FS), left ventricular end-diastolic diameter (LVEDD), and left ventricular end-systolic diameter (LVESD) were calculated via M-mode imaging with a 30 MHz transducer (SiliconWave 60; Cona Medical Technology Co., Ltd., Suzhou, Jiangsu Province, China).

**Ischemic infarction size assessment**

Mice were sacrificed and subjected to cardiac perfusion to harvest the entire heart tissue. The heart was rinsed in ice-cold phosphate-buffered saline (PBS), then removed and blotted dry with filter paper. It was placed in a 6 cm Petri dish and frozen at −20 °C for 20 min. The frozen heart tissue was rapidly sliced into 2 mm-thick coronal sections. These sections were stained with 1% 2,3,5-triphenyltetrazolium chloride (TTC) solution. Images were acquired and analyzed using ImagePro Plus 6.3 software. Total infarct size (%) was calculated as the ratio of infarct area to the total area of the heart coronal sections.

**Histological analysis**

Collagen fibers were analyzed by Masson trichrome staining and Sirius staining. Myocardial tissue samples were collected and fixed in 4% paraformaldehyde. Subsequently, the heart tissues were sectioned into short axis pieces. This included processing embedded tissue sections, removing paraffin with two changes of xylene, and rehydration using a gradient of ethanol concentrations (100, 95, 80, and 70%). The staining was performed according to the Masson staining kit (Servicebio, Wuhan, Hubei Province, China), Hematoxylin and Eosin (HE) Staining Kit (Servicebio, China) and Sirius Red Staining Kit (Servicebio, China) instructions. The area of tissue fibrosis was subsequently visualized utilizing a fluorescence microscope.

**Immunohistochemical staining**

IHC staining commenced with the dewaxing and rehydration of the paraffin sections, followed by heat-induced antigen retrieval in 0.1 M citrate buffer solution at 94 °C for 20 min, with subsequent cooling to room temperature. Subsequently, the sections were incubated in the dark at room temperature for 25 min with 3% hydrogen peroxide to inhibit endogenous peroxidase activity. Following a 30 min room temperature block with 3% bovine serum albumin (BSA), the sections were incubated overnight at 4 °C with primary antibodies, including MTFP1 antibody (proteintech, Cat#:14257-1-AP; dilution ratio 1:200). After washing, the sections were exposed to the secondary antibody, goat anti-rabbit IgG HRP (Servicebio, Cat#: GB23303, China), at room temperature for 90 min. Following additional washing steps, the sections were stained with 3,3′-diaminobenzidine and counterstained with hematoxylin. Images of the stained sections were captured using the Olympus fluorescence microscope (BX63, Tokyo, Japan), and subsequent image analysis was performed using ImageJ software (2.14.0/1.54f).

**Gelatin zymography**

According to other literature reports [25], myocardial tissue samples from each group were homogenized in ice-cold lysis buffer and centrifuged at 12,000× *g* for 10 min at 4 °C to obtain the supernatant. Protein concentrations were determined using the BCA assay, and equal amounts of protein from each sample were adjusted to the same concentration. An 8% SDS-PAGE gel containing 0.1% gelatin was prepared. After electrophoresis, the gels were washed twice with 2.5% Triton X-100 to remove SDS and then incubated in substrate buffer (50 mmol/L Tris-HCl, pH 7.5, 5 mmol/L CaCl_2_, 0.02% NaN_3_) for 24 h at 37 °C. Following incubation, the gels were stained with 0.5% Coomassie Brilliant Blue R-250 and destained until clear bands representing gelatinolytic activity were visible against a dark blue background. Areas of lysis corresponding to pro-MMP9 and active MMP9 were quantified by densitometry.

**Detection of intracellular ROS generation and mitochondrial ROS production**

H9c2 cells were cultured on glass coverslips in 35 mm dishes and subjected to hypoxia (1% O_2_, 5% CO_2_, 94% N_2_) for 12 h. Intracellular ROS levels were assessed using the fluorescent probe DCFH-DA (10 µM; Beyotime, Shanghai, China). After incubation with the probe for 20 min at 37 °C in the dark, cells were washed twice with PBS and immediately observed under a fluorescence microscope (Olympus, Japan). Images were acquired using identical exposure settings, and fluorescence intensity was quantitatively analyzed with ImageJ software. For each group, three independent experiments were performed (n = 3), and representative images are shown with a scale bar of 200 μm.

**Statistical analysis**

All experimental data are presented as percentile distributions or as mean  ±  s.e.m., as indicated in the figure legends. Each experiment was independently repeated at least three times with reproducible results. For comparisons between two groups, an unpaired two-tailed Student’s *t*-test was used. For comparisons among more than two groups, one-way ANOVA followed by Tukey’s multiple-comparisons test was performed. Statistical analyses were conducted using GraphPad Prism 9 (GraphPad Software, San Diego, CA, USA) or Microsoft Excel (Microsoft, Redmond, WA, USA). A *p* value < 0.05 was considered statistically significant. Exact n values are provided in the figure legends. Sample sizes were chosen based on prior studies and standard practice in the field; no statistical methods were used to predetermine sample size. Randomization and blinding were not applied unless otherwise stated.

## 3. Results

### 3.1. DE-MRGs Is Mainly Involved in Mitochondrial-Related Functions and Pathways

**Figure 1:**
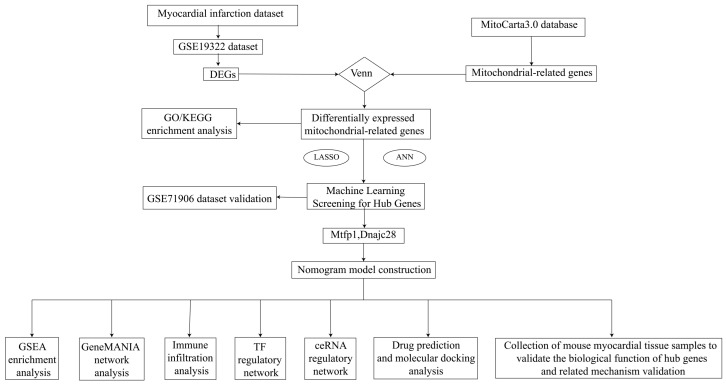
Flowchart of analysis. LASSO, Least Absolute Shrinkage and Selection Operator; ROC, receiver operating characteristics; KEGG, Kyoto Encyclopedia of Genes and Genomes; GO, Gene Ontology; ANN, artificial neural network; GSEA, Gene Set Enrichment Analysis; IHC, immunohistochemistry; RT-QPCR, quantitative real-time polymerase chain reaction; AUC, the area below the curve; MTFP1, mitochondrial fission process 1; DNAJC28, DnaJ heat shock protein family (Hsp40) member C2.

**Figure 2:**
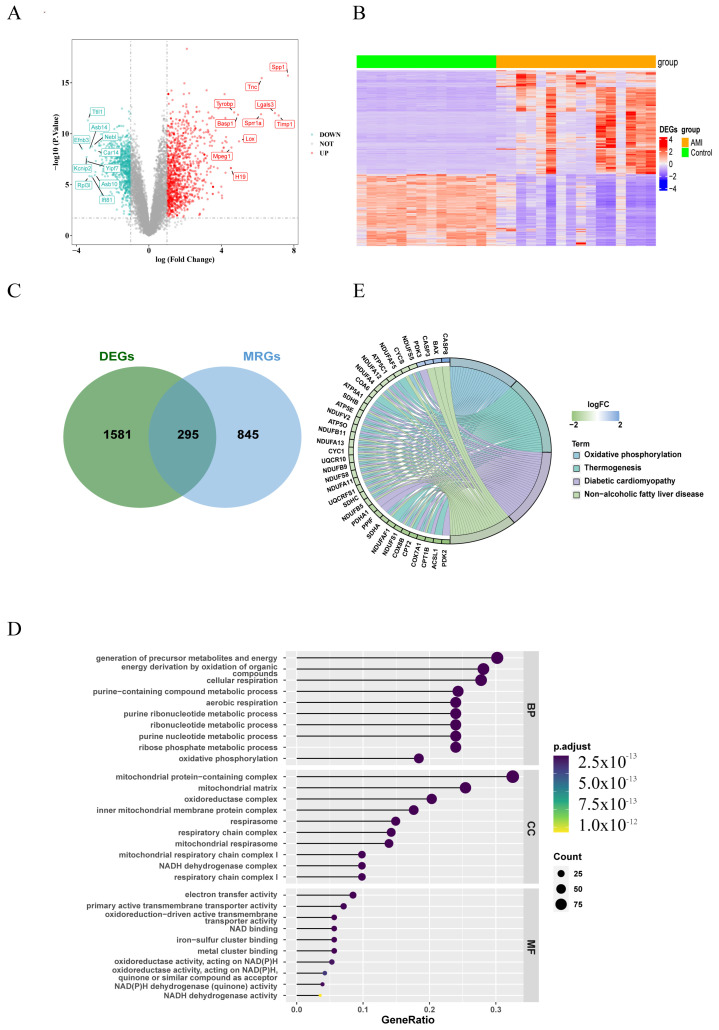
Differentially expressed genes related to mitochondria (DE-MRGs). (**A**) Volcano plot of 1876 DEGs (differentially expressed genes) between AMI and control samples. Red dots represent significantly upregulated genes, while green dots indicate significantly downregulated genes. The dashed line represents the statistical significance threshold. The horizontal reference line indicates the *p*-value threshold: −Log10(0.05) = 1.3, and the vertical reference line indicates the fold change (FC) threshold: log2FC = ±1. (**B**) Heatmap of 1876 DEGs between AMI and control samples (red and blue represent DEGs with upregulated and downregulated gene expression, respectively). (**C**) Venn diagram showing overlapping genes between DEGs and MRGs (mitochondria-related genes). (**D**) Gene Ontology (GO) enrichment analysis of 295 differentially expressed mitochondria-related genes (DE-MRGs). (**E**) Kyoto Encyclopedia of Genes and Genomes (KEGG) enrichment analysis of 295 DE-MRGs. BP: Biological Process; CC: Cellular Component; MF: Molecular Function.

The whole design flowchart of this research is presented in Figure 1. In total, 1876 DEGs were obtained between the AMI samples and the control samples, of which 1108 genes were upregulated and 768 genes were downregulated (Figure 2A,B). Subsequently, 295 DE-MRGs were screened by overlapping DEGs and MRGs (Figure 2C). For DE-MRGs, GO and KEGG were performed to know features of the organism’s biological composition and functional characteristics. A total of 425 GO BPs, 76 GO CCs, 149 GO MFs and 205 KEGG pathways were enriched. Specifically, the DE-MRGs were mainly enriched to GO entries such as oxidative phosphorylation, mitochondrial protein-containing complex and NAD binding, etc. (*p* value < 0.05) (Figure 2D). KEGG enrichment results included thermogenesis, diabetic cardiomyopathy and non-alcoholic fatty liver disease, etc. (*p* value < 0.05) (Figure 2E). These pathways and activities are associated with cellular energy production and mitochondrial function and may play an important role in the pathogenesis of acute myocardial infarction.

### 3.2. Identify DNAJC28 and MTFP1 as Biomarkers and Construct Nomograms

**Figure 3:**
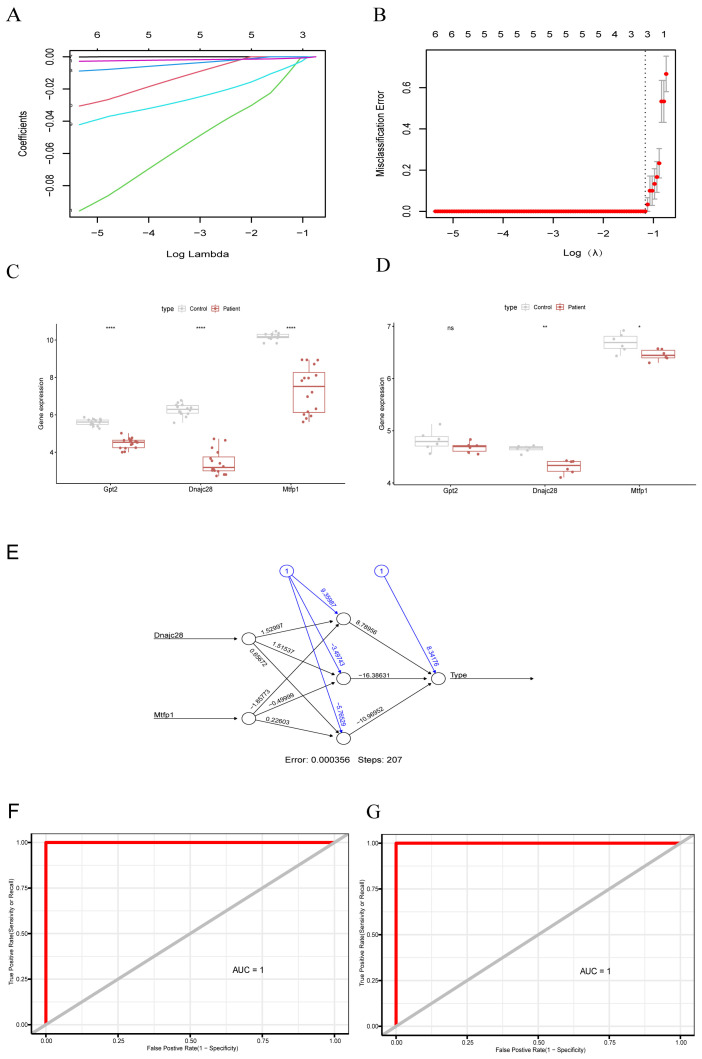
Identification of key mitochondria-related Genes using machine learning approaches. (**A**) Cross-validation plot of the LASSO (Least Absolute Shrinkage and Selection Operator) model. The horizontal axis represents log(Lambda), and the vertical axis represents the local likelihood deviation. Each colored line in the graph corresponds to the trajectory of the coefficient for a candidate gene as λ varies. The numbers labeled at the top of the graph indicate the count of genes with non-zero coefficients at different λ values. (**B**) Coefficient profile plot of LASSO regression. (**C**) Expression box diagram of biomarkers in dataset GSE19322. (**D**) Expression box diagram of biomarkers in dataset GSE71906. *p*  <  0.05 (*), *p*  <  0.01 (**), and *p*  <  0.0001 (****). (**E**) Artificial neural network (ANN) diagram of the identified biomarkers. (**F**) ROC curve analysis of biomarkers in the GSE19322 dataset. (**G**) ROC curve analysis of biomarkers in the GSE71906 dataset. The sensitivity value is plotted on the *Y* axis and the false positive (1-specific) value is plotted on the *X* axis. ROC: receiver operating characteristic, AUC: area under the curve.

The results of LASSO regression analysis showed that the model with the smallest error was obtained at lambda.min of 0.312, and three characterized genes (*GPT2*, *DNAJC28*, *MTFP1*) were screened out (Figure 3A,B). The results of expression validation showed that the *DNAJC28* and *MTFP1* genes exhibited significant differences and consistent expression trends in both the GSE19322 and GSE71906 datasets. The expression of *GPT2* showed insignificant differences in the GSE71906 dataset, but the expression trend was consistent with the GSE19322 dataset (Figure 3C,D). Immediately after that, we validated the diagnostic ability of the biomarkers using the ANN model, and the results showed that biomarkers could distinguish diseases and samples well (Figure 3E). Next, we validated the predictive performance of the biomarkers using ROC curves in both the GSE19322 and GSE71906 datasets. In both datasets, the results showed AUC = 1, which proved that the biomarker had a good predictive performance (Figure 3F,G).

**Figure 4:**
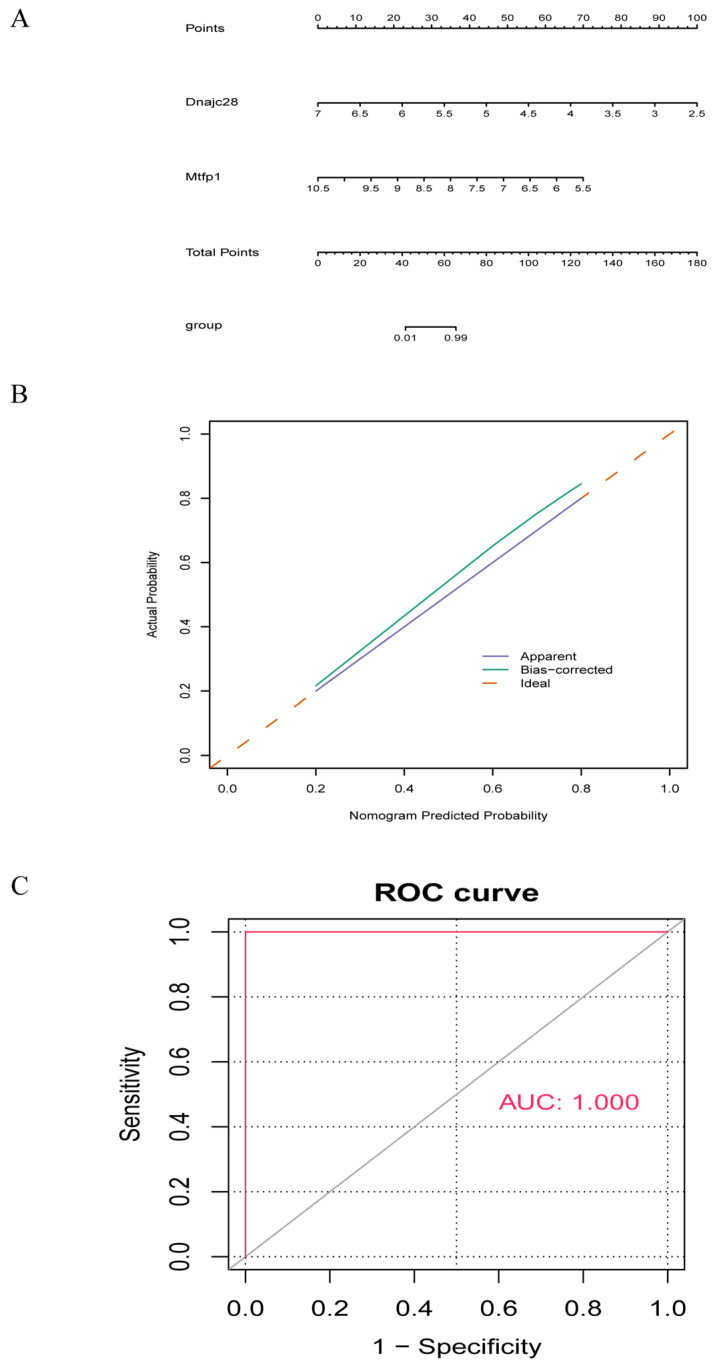
Nomogram model of biomarkers. (**A**) Nomogram model of the biomarkers. (**B**) Calibration curve evaluating the predictive accuracy of the nomogram model. (**C**) DCA evaluating the clinical utility of the nomogram model. The solid red line represents ROC curves for the biomarker based diagnostic model in the training set GSE19322 and validation set; the dashed gray line represents the diagonal reference (representing random guess performance). AUC = 1.000 represents an area under the curve of 1.000. ROC, receiver operating characteristic curve; AUC, area under the curve; DCA, decision curve analysis.

A nomogram model was constructed based on the biomarkers obtained from the above analysis (Figure 4A). The calibration curve showed that the error between the actual disease risk and the predicted risk is very small, demonstrating that the nomogram model has a high prediction accuracy for the disease samples (Figure 4B). From the ROC curve, it could be seen that the AUC value of the nomogram model reached one, which further illustrated the effectiveness of the nomogram model (Figure 4C).

### 3.3. GeneMANIA Network Analysis and Functional Enrichment Analysis

**Figure 5:**
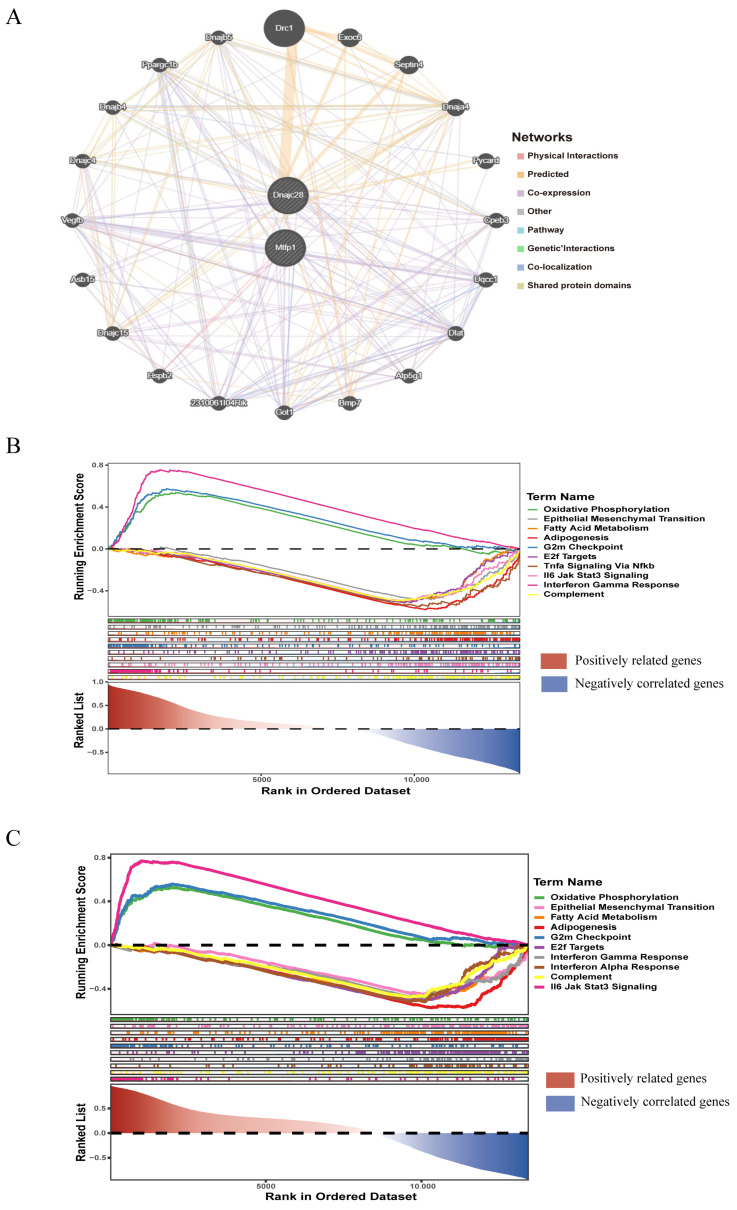
GeneMANIA network analysis and single-gene GSEA enrichment analysis. (**A**) GeneMANIA interaction network of key genes. (**B**) GSEA enrichment of DNAJC28. (**C**) GSEA enrichment of MTFP1. The red area (left region) represents the region where genes positively correlated with the target phenotype are enriched. At the left side of the ranked gene list (the end with smaller rank values), genes highly positively correlated with AMI are concentrated. If the peak of the enrichment score curve for a gene set falls on the left side, it indicates that the gene set is primarily enriched among positively correlated genes, potentially promoting the disease process. The blue area (right region) represents the region where genes negatively correlated with the target phenotype are enriched. At the right side of the ranked gene list (the end with larger rank values), genes highly negatively correlated with AMI are concentrated. If the peak of the enrichment score curve for a gene set falls on the right side, it indicates that the gene set is primarily enriched among negatively correlated genes, potentially inhibiting the disease process.

To learn about the functions and related proteins of these biomarkers, we utilized the GeneMANIA database to construct the GeneMANIA network and obtained 20 related genes (Figure 5A). For example, *DNAJC28* could physically interact with Exoc6; they were involved in predicted function. *MTFP1* could physically interact with Uqcc1; they were involved in co-expression function. The enrichment results of *DNAJC28* and *MTFP1* in the background gene set were selected to show the TOP10 results of enrichment significance for each gene. *DNAJC28* was enriched in oxidative phosphorylation, epithelial–mesenchymal transition, and E2f targets, etc. (Figure 5B). MTFP1 participated in TNF-α signaling, fatty acid metabolism, IFN-γ response, etc., through -NF-κB (Figure 5C).

### 3.4. Biomarkers and Immune Cell Infiltration

**Figure 6:**
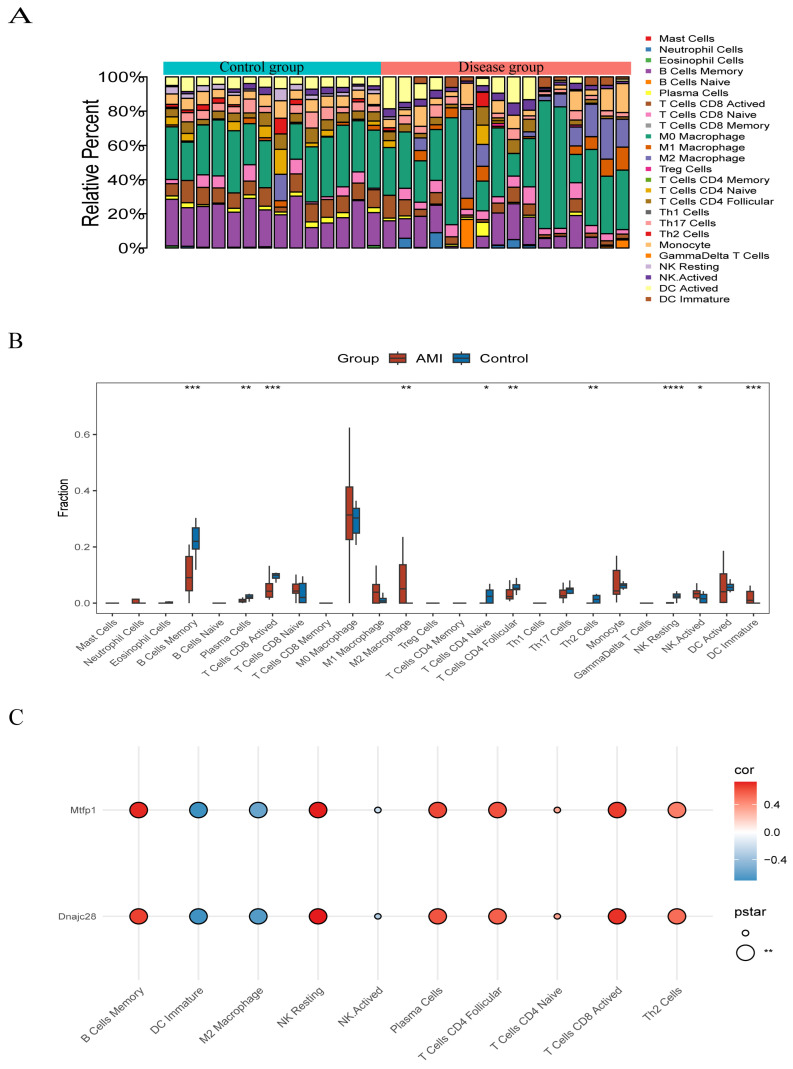
Biomarkers and immune cell infiltration. (**A**) Stacked bar plot of immune cell composition. (**B**) Box plot of immune cell infiltration levels. (**C**) Heatmap showing correlations between differential immune cells and biomarkers. Significance levels were defined as: *p*  <  0.05 (*), *p*  <  0.01 (**), *p*  <  0.001 (***), and *p*  <  0.0001 (****).

Immune cells infiltrated in pathological tissues are most likely to serve as drug targets to improve the survival rate of patients. In the disease and control samples, we obtained the proportions of 22 kinds of immune cells (*p* < 0.05); M0 Macrophage accounted for a large proportion in both samples (Figure 6A). The results showed that there were significant differences in 10 kinds of immune cells between the disease group and the control group, such as B Cells Memory, DC Immature, NK Resting, etc. (Figure 6B). Spearman correlation analysis showed eight significant correlations between the differential immune cells and the biomarkers, six of which were positive and two were negative. Specifically, B Cells Memory was significantly positively correlated with MTFP1 and DC Immature was significantly negatively correlated with DNAJC28 (Figure 6C).

### 3.5. Two Network Analysis and Five Targeted Drugs for Biomarkers

**Figure 7:**
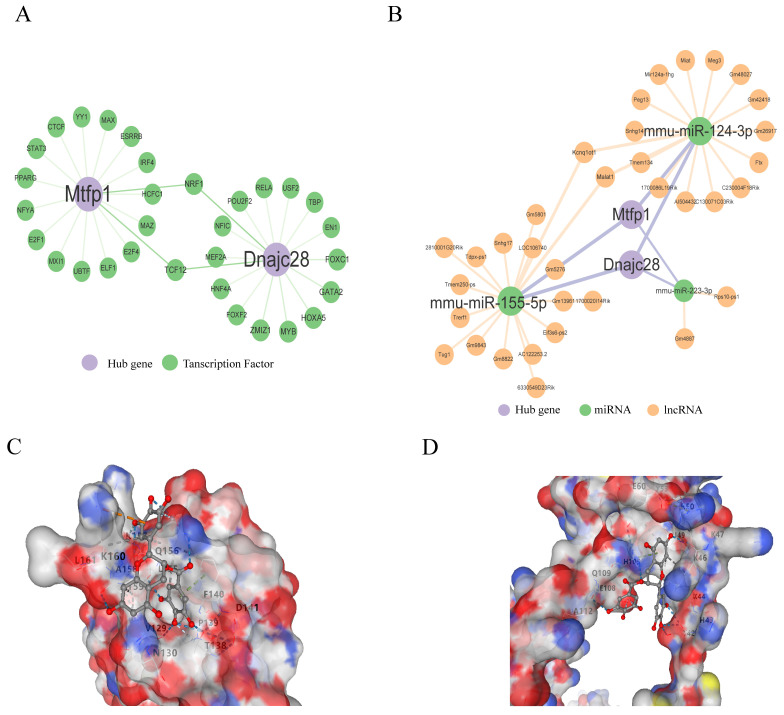
Dual network analyses and five targeted drugs for biomarkers. (**A**) Transcription factor-gene regulatory network, showing predicted upstream TFs targeting *MTFP1* and *DNAJC28*; each green node represents a TF, with purple edges denoting regulatory interactions. (**B**) Competing endogenous RNA (ceRNA) regulatory network for *MTFP1* and *DNAJC28*. Orange nodes represent lncRNAs, green nodes represent miRNAs, and purple edges denote miRNA–mRNA and lncRNA–miRNA interactions. Key interactions include mmu-miR-155-5p and mmu-miR-124-3p co-regulating both target genes. A Vina score lower than −5 kcal/mol indicates favorable binding affinity. Notably, epigallocatechin gallate showed the strongest docking affinity with both targets (−7.1/−7.6 kcal/mol). (**C**,**D**) Predicted docking conformation of epigallocatechin gallate (EGCG) within the MTFP1 and DNAJC28 binding pocket. Electrostatic surface rendering highlights hydrogen bond interactions and hydrophobic contacts.

The TFs of biomarkers were predicted based on three databases, and 31 TFs for two biomarkers were obtained (Figure 7A), such as YY1-MTFP1, MAX-MTFP1, MAZ-MTFP1, EN1-DNAJC28, and TBP-DNAJC28, USF2-DNAJC28, suggesting that these genes may be transcriptionally regulated in stress and injury responses related to AMI. In addition, three miRNAs were predicted to target both *MTFP1* and *DNAJC28*, and 34 lncRNAs were identified as interacting with these miRNAs through a ceRNA regulatory axis. A representative ceRNA pathway includes Gm4887-mmu-miR-223-3p-MTFP1 and Gm9843-mmu-miR-155-5p-DNAJC28, forming potential post-transcriptional regulatory loops (Figure 7B). These interactions suggest the existence of complex lncRNA-miRNA-mRNA competing endogenous RNA networks that may modulate mitochondrial function and fibrosis during AMI.

To explore therapeutic potentials, five small-molecule compounds targeting MTFP1 and DNAJC28 were identified through drug signature enrichment analysis using the Enrichr platform (DSigDB database) (Table 1). Notably, raloxifene HL60 UP (combined score: 175.24), meclofenoxate HL60 UP (165.72), and doxycycline HL60 UP (164.03) were among the top candidates predicted to modulate DNAJC28 expression. Potassium chromate (CTD00001284) and epigallocatechin gallate (EGCG, CTD00002033) were found to target both MTFP1 and DNAJC28. To assess the binding affinity of these drugs with the protein targets, 3D protein structures of MTFP1 and DNAJC28 were retrieved from the Protein Data Bank (PDB). The drug structures were obtained from the PubChem database, and molecular docking was performed using CB-Dock, an online blind docking tool. A docking score below −5.0 kcal/mol was considered indicative of a strong binding interaction. The results showed that EGCG exhibited the strongest binding affinity, with docking scores of −7.1 and −7.6 kcal/mol for MTFP1 and DNAJC28, respectively (Table 2). The docking conformations highlight the key interacting residues and the electrostatic surfaces of the binding pockets, supporting the potential of EGCG as a lead compound for therapeutic modulation of mitochondrial dynamics and ECM remodeling in AMI (Figure 7C,D).

### 3.6. Expression of DNAJC28 and MTFP1 in Mouse Myocardial Infarction Model and Myocardial Cell Hypoxia Model In Vitro

**Figure 8:**
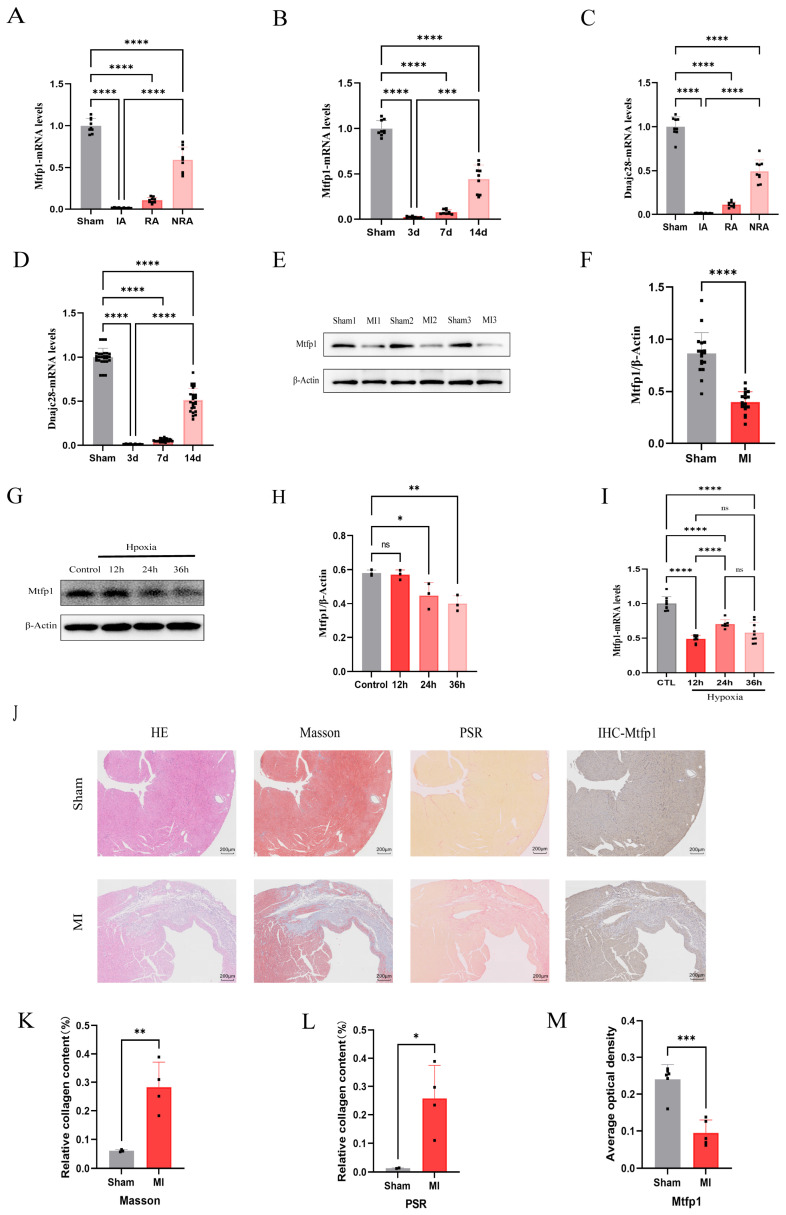
Expression profiles of biomarkers MTFP1 and DNAJC28 in vivo and in vitro models. (**A**) mRNA expression levels of MTFP1 in different cardiac regions of C57/BL6 male mice randomly assigned to sham or MI groups (n = 3). (**B**) Temporal mRNA expression patterns of MTFP1 at different post-MI timepoints. (**C**) mRNA expression levels of DNAJC28 in different cardiac regions. (**D**) Temporal mRNA expression patterns of DNAJC28 at different post-MI timepoints. (**E**,**F**) Protein expression of MTFP1 in sham and MI groups. (**G**,**H**) MTFP1 protein expression in cardiomyocytes under normoxia vs. hypoxia at indicated durations. (**I**) MTFP1 mRNA expression in cardiomyocytes during hypoxic exposure. (**J**) Representative images of HE staining (scale bar = 200 μm). Masson’s trichrome (scale bar = 200 μm). Picrosirius red staining (scale bar = 200 μm). MTFP1 immunohistochemistry (scale bar = 200 μm) in sham vs. MI hearts. (**K**,**L**) Quantification of Collagen Content by Masson and Sirius Red Staining expressed as a percentage. (**M**) Mean optical density of MTFP1 in sham operation group and myocardial infarction group. All experiments were conducted in triplicate with three independent biological replicates per group. Data are presented as mean ± SEM. Statistical analysis was performed using unpaired two-tailed Student’s *t*-test for two-group comparisons and one-way ANOVA followed by Tukey’s post hoc test for multiple-group comparisons. Significance levels were defined as: *p*  <  0.05 (*), *p*  <  0.01 (**), *p*  <  0.001 (***), and *p*  <  0.0001 (****).

**Figure 9:**
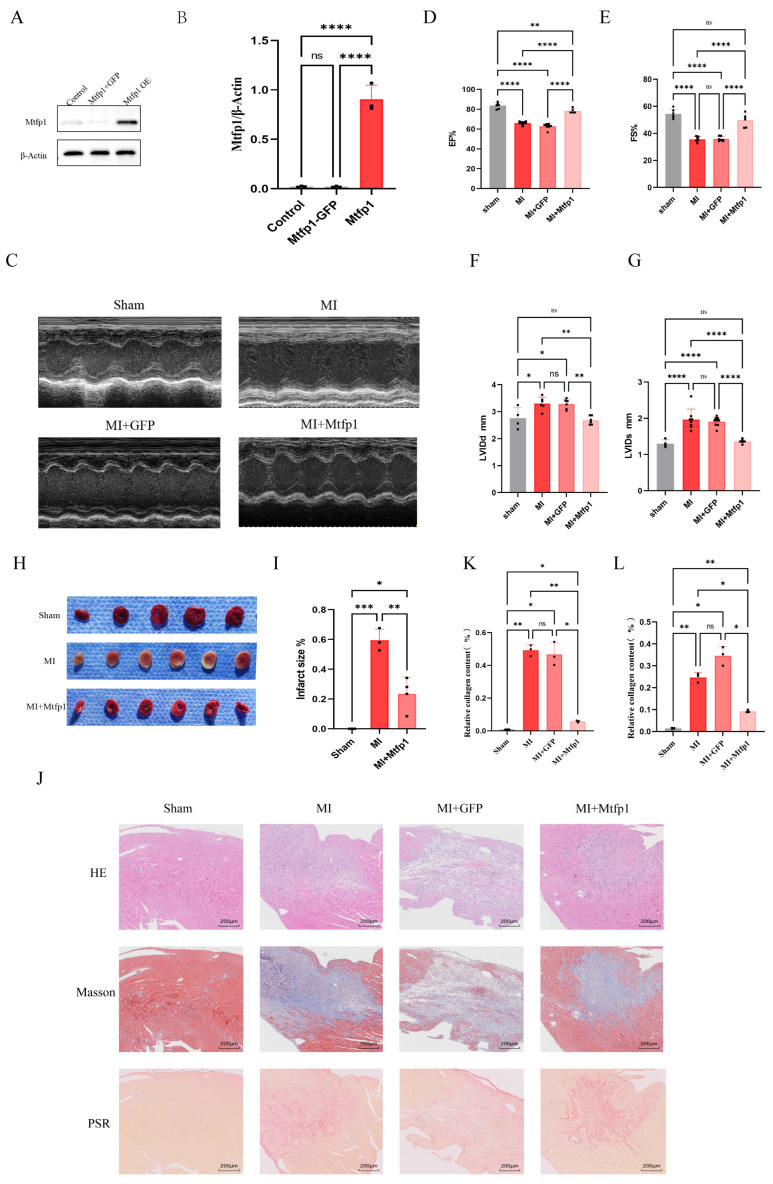
MTFP1 overexpression ameliorates cardiac function, reduces infarct size, and mitigates post-MI fibrosis. (**A**,**B**) Validation of MTFP1 overexpression. C57/BL6 male mice were randomly allocated into: control group (n = 3), GFP group (n = 3), MTFP1 overexpression group (n = 3). MTFP1 expression levels were quantified to confirm successful overexpression. (**C**–**G**) Echocardiographic Assessment of Cardiac Function. Mice were randomized into: sham group (n = 3), MI group (n = 3), MI + GFP group (n = 3), MI + MTFP1 group (n = 3). Parameters evaluated: Ejection fraction (EF%), fractional shortening (FS%), left ventricular end-diastolic diameter (LVEDD; mm), left ventricular end-systolic diameter (LVESD; mm). (**H**,**I**) Infarct size quantification by TTC staining. Experimental groups: Sham (n = 3), MI (n = 3), MI + MTFP1 (n = 3). (**J**–**L**) Histopathological evaluation. Groups analyzed: Sham (n = 3), MI (n = 3), MI + GFP (n = 3), MI + MTFP1 (n = 3). Staining performed: HE staining (scale bar = 200 μm), Masson’s trichrome (scale bar = 200 μm), Picrosirius red (scale bar = 200 μm). All experiments were independently repeated three times. All experiments were conducted in triplicate with three independent biological replicates per group. Data are presented as mean ± SEM. Statistical analysis was performed using one-way ANOVA followed by Tukey’s post hoc test for multiple-group comparisons. Significance levels were defined as: *p*  <  0.05 (*), *p*  <  0.01 (**), *p*  <  0.001 (***), and *p*  <  0.0001 (****).

A mouse model of myocardial infarction was established, and the mRNA expressions of *DNAJC28* and *MTFP1* were detected in the infarcted area, the infarcted edge area and the non-infarcted area of the mouse heart. At the same time, the mRNA levels were detected in the infarcted area at the 3rd, 7th and 14th days of myocardial infarction. The results showed that the expression of *MTFP1* and *DNAJC28* was the lowest in the infarcted area, followed by the infarcted edge area and the highest in the non-infarcted area (Figure 8A,C). At the same time, the expression was the lowest at 3 days of myocardial infarction, followed by 7 days, and the expression was restored by 14 days (Figure 8B,D). The expression of MTFP1 protein in the heart of mice with myocardial infarction for 7 days was detected, and the results showed that the expression in the myocardial infarction group was significantly lower than that in the sham operation group (Figure 8E,F). Furthermore, an in vitro hypoxia model of H9c2 cell was established, and the protein and mRNA levels of *MTFP1* were detected. The results showed that the protein and mRNA levels of *MTFP1* decreased significantly after 24 h and 36 h of hypoxia (Figure 8G,H and Figure 9I). HE staining, Masson staining, Sirius red staining and immunohistochemical staining were performed on the tissue sections of mice with myocardial infarction and sham operation group. The results showed that compared with the sham operation group, the myocardial infarction group had significant inflammatory infiltration (Figure 8J), fibrosis increased significantly (Figure 8J–L), and the expression of MTFP1 in the infarction group decreased significantly compared with the sham operation group (Figure 8J,M).

### 3.7. MTFP1 Overexpression Improves Cardiac Function, Reduces Infarct Size, and Attenuates Fibrosis

Following transfection with AAV9-*MTFP1* overexpression vectors, cardiac tissue was harvested for validation of MTFP1 expression (Figure 9A,B). Myocardial infarction surgery was performed on mouse transfected with aav9-MTFP1 overexpressing virus. The heart ultrasound was detected seven days after the operation. The results showed that the cardiac function after myocardial infarction was significantly improved after overexpression of MTFP1 (Figure 9C–G). Further, we performed TTC staining, and the results showed that the area of myocardial infarction was significantly improved after overexpressing MTFP1 (Figure 9H,I). The myocardial tissue sections of mouse were stained with HE, Masson’s trichrome and picrosirius red. The results showed that overexpression of MTFP1 significantly improved fibrosis after myocardial infarction (Figure 9J–L).

### 3.8. MTFP1 Regulates the p-DRP1/MMP9/TIMP1 Axis to Restore ECM Remodeling in Post-MI Hearts and Hypoxia-Induced Cardiomyocytes

**Figure 10:**
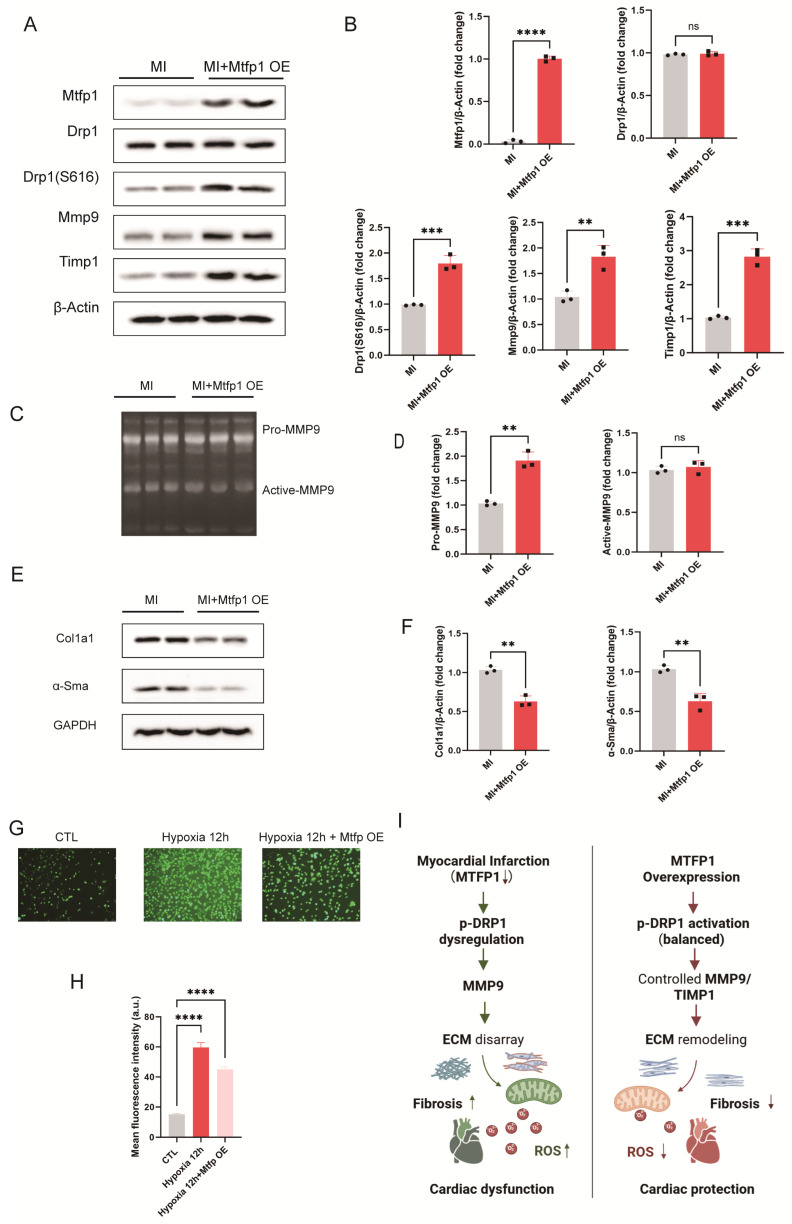
MTFP1 restores mitochondrial fission–fusion balance and ECM remodeling via p-DRP1/MMP9/TIMP1 axis in hypoxia-induced cardiomyocytes. (**A**,**B**) Western blot analysis of myocardial tissue from MI and MI + MTFP1 overexpression (OE) groups assessing MTFP1, DRP1, p-DRP1 (Ser616), MMP9, and TIMP1 expression. Quantification of protein expression is shown (n = 3/group). (**C**,**D**) Gelatin zymography of myocardial tissue showing pro-MMP9 and active MMP9 levels, with corresponding quantification (n = 3/group). (**E**,**F**) Western blot analysis of fibrosis-associated proteins COL1A1 and α-SMA in myocardial tissue and corresponding quantification (n = 3/group). (**G**,**H**) ROS levels in hypoxia-induced H9C2 cell detected by DCFH-DA fluorescence staining and quantitative fluorescence intensity analysis (n = 3/group; scale bar = 200 μm). (**I**) Schematic illustration of the proposed mechanism: MTFP1 overexpression restores mitochondrial dynamics and ECM remodeling by regulating the p-DRP1/MMP9/TIMP1 signaling axis, thereby attenuating fibrosis and oxidative stress while improving cardiac function. All experiments were conducted in triplicate with three independent biological replicates per group. Data are presented as mean ± SEM. Statistical analysis was performed using unpaired two-tailed Student’s *t*-test for two-group comparisons and one-way ANOVA followed by Tukey’s post hoc test for multiple-group comparisons. Significance levels were defined as: *p*  <  0.01 (**), *p*  <  0.001 (***), and *p*  <  0.0001 (****).

To further elucidate the mechanisms underlying the cardioprotective effects of MTFP1, we examined its influence on mitochondrial fission and ECM remodeling in myocardial tissue and hypoxia-induced cardiomyocytes. In myocardial tissue, Western blot analysis showed that MTFP1 overexpression significantly elevated p-DRP1 (Ser616) levels without altering total DRP1 expression compared to the MI group. Additionally, MMP9 expression exhibited a moderate increase, accompanied by a pronounced upregulation of TIMP1, indicating that MTFP1 may preserve ECM homeostasis through TIMP1-mediated regulation of MMP9 activity (Figure 10A,B). Gelatin zymography revealed that MTFP1 overexpression markedly increased pro-MMP9 levels in infarcted myocardium, whereas active MMP9 levels remained largely unchanged (Figure 10C,D). These findings suggest that although MMP9 synthesis was enhanced, its activation was effectively restrained—likely due to concurrent TIMP1 induction—thereby maintaining balanced ECM proteolysis. Correspondingly, fibrosis-associated proteins COL1A1 and α-SMA were significantly reduced in MTFP1 overexpressing hearts relative to MI alone (Figure 10E,F), underscoring the antifibrotic effects of MTFP1 in post-infarct myocardium. Moreover, ROS levels, assessed by DCFH-DA staining, were markedly increased under hypoxia but substantially reduced following MTFP1 overexpression (Figure 10G,H), indicating mitigation of oxidative stress. Collectively, these findings demonstrate that MTFP1 mitigates myocardial fibrosis and oxidative injury by modulating the p-DRP1/MMP9/TIMP1 axis, thereby restoring ECM remodeling and improving mitochondrial homeostasis in both infarcted myocardium and hypoxia-challenged cardiomyocytes (Figure 10I).

## 4. Discussion

AMI is characterized by myocardial ischemia/hypoxia and subsequent cardiomyocyte necrosis, and mitochondrial dysfunction is a central contributor to its pathogenesis [26,27,28]. In this study, we integrated bioinformatics and experimental validation to identify mitochondrial function-related genes involved in AMI. From the GSE19322 dataset, we identified 295 mitochondrial-related DEGs, which were mainly enriched in oxidative phosphorylation, mitochondrial metabolism, and energy production pathways, supporting the importance of mitochondrial homeostasis in AMI progression.

Using LASSO regression, we further screened two characteristic genes, *MTFP1* and *DNAJC28*, and established an optimal classification model. Experimental validation (IHC, RT-qPCR, and Western blot) confirmed that MTFP1 was significantly downregulated in AMI, particularly in the infarcted myocardium, suggesting its potential involvement in myocardial injury and post-infarction remodeling. Importantly, while mitochondrial dysfunction, DRP1-related signaling, and ECM remodeling have each been implicated in post-MI repair; the specific contribution of MTFP1 as a potential upstream integrative node linking these processes has remained unclear. Thus, the novelty of the present study lies in prioritizing MTFP1 through a mitochondria-focused screening strategy and providing in vivo functional evidence supporting its role in post-infarction remodeling.

MTFP1 is a mitochondrial inner membrane protein associated with mitochondrial fission, apoptosis, and metabolic homeostasis. Previous studies have reported context-dependent roles of MTFP1: in some settings, MTFP1 overexpression promotes mitochondrial fission, ROS production, and apoptosis, whereas in others, MTFP1 deficiency impairs cell viability and mitochondrial function [14,29,30,31,32,33,34]. In the heart, cardiomyocyte-specific loss of MTFP1 causes progressive dilated cardiomyopathy and mitochondrial abnormalities, indicating that appropriate MTFP1 expression is required for cardiac mitochondrial integrity [3]. Accordingly, our findings should be interpreted as extending this context-dependent framework to AMI remodeling, rather than contradicting prior observations in other disease models.

Mechanistically, we focused on mitochondrial dynamics and ECM remodeling. MTFP1 overexpression significantly increased p-Drp1 (Ser616) without changing total Drp1, suggesting regulation at the activation level rather than expression abundance. In parallel, MTFP1 overexpression increased pro-MMP9 but did not enhance activated MMP9, which may be related to concomitant TIMP1 upregulation. Together, these results suggest that MTFP1 may help coordinate mitochondrial dynamics and ECM turnover, thereby favoring more organized remodeling after MI. We note that the involvement of DRP1 signaling and MMP9/TIMP1-associated ECM regulation in MI remodeling is broadly consistent with the existing literature; therefore, these findings are best viewed as an initial mechanistic framework that places MTFP1 within an established pathophysiological axis, rather than as definitive proof of causality.

From a clinical perspective, our results may be relevant to post-infarction IHD phenotyping, in which myocardial viability and scar burden are critical determinants of recovery and remodeling. CMR offers an important clinical framework for assessing these tissue-level features together with ventricular function. Although our study did not directly evaluate CMR-derived viability or scar burden, the observed effects of MTFP1 overexpression (improved function, reduced infarct size, and attenuated fibrosis) suggest a potentially more favorable viability–scar profile. Future studies integrating MTFP1-targeted interventions with serial CMR assessment (including infarct/scar burden and viable myocardium) would help establish the translational significance of these findings [35].

DNAJC28 (DnaJ homolog subfamily C member 28), a member of the HSP40 family, was identified as another candidate gene in our model. Although evidence regarding DNAJC28 in cardiovascular disease is currently limited, HSP40 family proteins have been implicated in AMI-related processes and cardiac regeneration [36,37]. Our data showed downregulation of DNAJC28 in AMI, suggesting that it may participate in MI-associated pathological changes; however, its precise role requires further functional validation. Thus, the novelty of DNAJC28 in the present study is primarily at the candidate gene discovery/prioritization level, whereas its mechanistic role remains to be established.

To further characterize the potential functional context of the key genes, enrichment and GSEA analyses indicated close associations with oxidative phosphorylation, fatty acid metabolism, and EMT-related pathways. These results are consistent with the concept that mitochondrial metabolic remodeling and ECM-related phenotypic transitions are interconnected during AMI progression and repair [38,39,40,41]. This aspect of the study is largely confirmatory in nature, but it strengthens the biological plausibility of MTFP1/DNAJC28 as AMI-related mitochondrial candidates. We also constructed a ceRNA regulatory network and predicted three miRNAs (mmu-miR-124-3p, mmu-miR-155-5p, and mmu-miR-223-3p) potentially interacting with MTFP1 and DNAJC28, along with multiple literature-supported lncRNAs. Given the reported roles of these miRNAs in ischemic injury, inflammation, and angiogenesis [42,43,44,45], our predictions provide hypothesis-generating leads for future studies, but require experimental validation.

Because inflammation and immunity are critical in AMI pathogenesis [46,47,48,49,50,51], we also analyzed immune infiltration and observed significant differences in 10 immune cell types between AMI and control groups, including macrophages, neutrophils, NK cells, T cells, B cells, and dendritic cells. Correlation analysis further showed a positive association between memory B cells and MTFP1, and a negative association between immature dendritic cells and DNAJC28, suggesting that the immune microenvironment may be linked to the regulation of these genes during myocardial injury and repair. These immune analyses should also be interpreted as supportive/contextual findings that help position the candidate genes within the AMI microenvironment, rather than as direct mechanistic evidence.

Drug prediction analysis identified EGCG and potassium chromate as potential molecules targeting MTFP1 and DNAJC28, and molecular docking suggested favorable binding of EGCG to both proteins. Given the known antioxidant and antifibrotic effects of EGCG [52], these findings suggest a possible therapeutic direction for further validation in AMI. At present, this remains an in silico prediction and does not alter the main novelty claim of the study, which is centered on MTFP1 identification and in vivo functional validation.

This study has several limitations. First, experimental validation was more comprehensive for *MTFP1* than for *DNAJC28*. Second, the proposed mechanisms are primarily correlative and were not confirmed by loss-of-function or rescue experiments. Third, our conclusions are based mainly on animal models and bioinformatics analyses, and therefore require further clinical validation. Finally, although our data support placing MTFP1 within a p-Drp1/MMP9/TIMP1-related remodeling framework, the upstream–downstream causal hierarchy and cell type-specific effects remain to be clarified. In addition, although our histological and functional data indicate beneficial remodeling effects, we did not perform clinically translatable tissue characterization imaging (e.g., CMR-based assessment of myocardial viability and scar burden), which will be important for validating the translational relevance of MTFP1 in post-infarction IHD.

## 5. Conclusions

This study identifies *MTFP1* and *DNAJC28* as mitochondrial function-related candidate genes associated with AMI and provides experimental evidence consistent with a protective role of MTFP1 in post-infarction remodeling. While the involvement of mitochondrial dynamics and ECM remodeling in MI repair has been supported by prior studies, our work adds novelty by nominating MTFP1 as a candidate molecular link between these processes and by demonstrating the beneficial effects of MTFP1 restoration in vivo. Our findings suggest that MTFP1 may contribute to myocardial repair, at least in part, through regulation of mitochondrial dynamics and ECM homeostasis, thereby providing a basis for future mechanistic and translational studies in AMI. In contrast, the role of DNAJC28 should currently be considered a discovery-level finding that requires dedicated functional validation. Future studies integrating molecular interventions with clinically relevant imaging endpoints, particularly CMR-based viability and scar quantification, may help clarify the translational value of MTFP1 in post-MI risk stratification and remodeling-guided management.

## Figures and Tables

**Table 1:** Five candidate small-molecule compounds predicted to target MTFP1 and/or DNAJC28.

Term	*p*-Value	Combined Score	Genes
POTASSIUM CHROMATE CTD 00001284	0.01	170,581.13	MTFP1; DNAJC28
Epigallocatechin gallate CTD 00002033	0.01	160,785.61	MTFP1; DNAJC28
raloxifene HL60 UP	0.04	175.24	DNAJC28
meclofenoxate HL60 UP	0.04	165.72	DNAJC28
doxycycline HL60 UP	0.04	164.03	DNAJC28

**Table 2:** Molecular docking scores between candidate compounds and MTFP1/DNAJC28 proteins.

Term	Genes	Vina Score
POTASSIUM CHROMATE CTD 00001284	MTFP1; DNAJC28	−6.7/−7.0
Epigallocatechin gallate CTD 00002033	MTFP1; DNAJC28	−7.1/−7.6
raloxifene HL60 UP	DNAJC28	−7.3
meclofenoxate HL60 UP	DNAJC28	−5.6
doxycycline HL60 UP	DNAJC28	−7.2

## Data Availability

The original contributions presented in this study are included in the article. Further inquiries can be directed to the corresponding authors.
